# Exploring the influence of music on cognitive performance in female assembly line workers at a medical device manufacturing unit

**DOI:** 10.1371/journal.pone.0309555

**Published:** 2024-10-23

**Authors:** Melika Abbasi, Reza Esmaeili, Siamak Pourabdian, Mahnaz Shakerian

**Affiliations:** 1 Student Research Committee, Department of Occupational Health and Safety Engineering, School of Health, Isfahan University of Medical Sciences, Isfahan, Iran; 2 Department of Occupational Health and Safety Engineering, School of Health, Isfahan University of Medical Sciences, Isfahan, Iran; Shahrood University of Medical Sciences, ISLAMIC REPUBLIC OF IRAN

## Abstract

The significance of enhancing working conditions for the physical health and performance of workers, particularly female workers, underscores the need for research in this domain and the examination of interventions such as music. Previous studies have yielded diverse outcomes regarding the influence of music on individuals’ performance; hence, further research in this area appears imperative. The aim of this study is to explore the impact of music on the cognitive and task performance of female assembly operation operators. This study is an interventional (quasi-experimental) study that involved 81 participants from the female workforce of the medical equipment assembly unit in Isfahan, Iran. The evaluation encompassed task performance, working memory using N-Back test, sustained attention using continues performance test (CPT), degree of sleepiness, and mental fatigue using Flicker Fusion test, along with physiological parameters such as heart rate and blood oxygen level. Participants underwent testing both in the presence of classical music and in a condition without music playback. The provision of background music enhanced the workers’ sustained attention and working memory. It led to improved task performance and a reduction in drowsiness. Concerning physiological parameters, it resulted in a slight decrease in heart rate at the end of the work shift and a marginal increase in participants’ blood oxygen levels. Background music enhanced working memory (p-value = 0.001), sustained attention (p-value = 0.001), and improved the task performance of workers(p-value = 0.005). Additionally, likely due to increased relaxation, it led to a decrease in heart rate (p-value = 0.001) and an increase in blood oxygen levels (p-value = 0.016). Music also played a role in reducing participants’ sleepiness (p-value = 0.001).

## 1. Introduction

Industrial production faces numerous challenges that profoundly impact work in industrial environments. As a crucial component of the manufacturing process, assembly involves facilitating communication among parts, kits, assemblies, and components. Essentially, the assembly of segments is a production process wherein replaceable parts are sequentially added to the final product. Assembly workers contend with significant physical workloads. Moreover, the production line in question is a domain where the individual cognitive performance of workers significantly influences overall work performance and quality [1–4]. Cognitive load, in particular, may result in decreased worker performance, potentially leading to costly assembly errors, wasted time, and heightened frustration. Results indicate that both positive and negative effects on the cognitive performance of assemblers can be attributed to factors such as work design, scheduling, physical load requirements, teamwork, and external motivational influences [5, 6].

Cognitive performance in assembly work pertains to how well individual workers can detect, recognize, process, and interpret relevant signals from the assembly environment, and then make decisions that lead to the correct assembly of components. The necessity to complete tasks within a limited timeframe can introduce pressure and stress, which can cause workers to allocate some of their cognitive resources to monitoring the time rather than focusing entirely on the task at hand. Sustainable cognitive performance is defined as maintaining an optimal mental workload that keeps assemblers alert and engaged with their tasks, aligns with their skill level, and preserves a sense of control. This balance helps avoid excessive or insufficient mental strain, which can result in overwhelm, distraction, or a lack of focus [5]. So, Falck et al. demonstrated in their study that there is an interaction between assembly ergonomics and assembly complexity factors. They emphasized that both factors should be proactively considered to minimize assembly-related failures and associated action costs [2]. Also, Zhu et al. discuss operator choice complexity, where operators must select the correct parts and tools based on customer orders, often under time pressure. In paced assembly lines, high cognitive and physical demands frequently result in mistakes, quality issues, and other assembly-related failures [7]. And, Bläsing et al. found that varied manual assembly requires significant cognitive effort, with increased task complexity raising the risk of cognitive overload. They suggested that better information presentation and adaptable informational assistance systems can mitigate these risks [8].

One of the external motivational factors influencing cognitive performance in work environments is the impact of both favorable and unfavorable auditory stimuli. Unpleasant auditory stimuli, such as the effects of harmful noises in the work environment, have a detrimental effect on cognitive performance. This negative impact can manifest in impaired cognitive performance, notably through a significant increase in response time and reaction time for the operator [9]. On the contrary, pleasant auditory stimuli, specifically in the form of music, exhibit diverse effects on cognitive performance [10–12]. Studies have demonstrated that background music can positively influence people’s cognitive performance, enhancing aspects such as sustained attention, concentration, and memory [10, 11, 13].

Regarding repetitive and monotonous jobs, some studies have indicated that the lack of variety in highly monotonous tasks can result in decreased alertness and the onset of fatigue. In such cases, a stimulus like music is needed to maintain a high level of alertness and resist fatigue [12, 14]. Interestingly, relaxing auditory influences, such as classical music, may even have a positive impact on workers’ accuracy. Furthermore, prior musical experience can be beneficial for surgeons, who may be particularly affected by music or noise [15]. Research has also been conducted to explore the positive effect of music on memory, with results demonstrating enhanced memory performance in the presence of music [12]. Notably, some studies have revealed that listening to music can reduce stress across various fields [16–18]. Music interventions are also employed in the field of occupational medicine to alleviate workplace distress and enhance mood, performance, positive emotions as well as cognitive skills like attention and concentration [18–23].

In a separate study conducted in a hospital operating room, it was discovered that playing music could significantly alleviate emotional distress among rotating nurses and nurse anesthetists, as well as reduce the psychological burden on nurse anesthetists [24]. Additionally, a study involving 100 operating room workers revealed that a majority of respondents were aware of the benefits of music, including improvements in cognitive functions, happiness, and mood. Respondents noted that music makes surgeons more considerate and calmer, enhances work efficiency, and reduces stress responses. Among these respondents, 87% supported the idea of playing music during surgery [25]. Current data indicate that listening to relaxing music leads to significantly lower stress and anxiety levels compared to listening to stimulating music [26]. The results of another study demonstrated a moderate effect of music interventions on outcomes related to mental stress, encompassing emotional states such as mental worry, anxiety, restlessness, and nervousness. It was determined that, in addition to reducing physiological arousal, music also influences emotional states [27]. On the other hand, studies have explored the effects of music on people’s health, including blood pressure [28, 29].

Blood pressure varies based on activity; in stressful environments, blood pressure increases, while in calm and relaxed settings, blood pressure tends to decrease. In this regard, classical music therapy (Mozart) has a positive impact that can soothe the mind, and the heartbeat gradually synchronizes with the rhythm of the music, resulting in lowered blood pressure. Classical music (Mozart) features a slow beat that aligns with the heart rhythm of adults and can stimulate alpha waves in the brain [29]. Previous studies have explored the relationship between music and people’s heart rate. Some studies have demonstrated a decrease in heart rate while listening to relaxing music [30, 31], while exciting music has been shown to increase heart rate [32]. Another study investigating the effects of music therapy revealed that healthy/asymptomatic elderly men experienced a significant reduction in systolic blood pressure, diastolic blood pressure, pulse rate, and respiration/breathing rate when exposed to music therapy intervention (listening to Raga Todi) for 30 days, particularly during morning daylight hours [28]. In past studies examining the impact of music on cognitive performance, most results indicate a positive effect of music on people’s cognitive performance [13, 25]. Researchers have demonstrated a positive relationship between music in the workplace and task performance, as well as various components of employee performance [33].

While most studies have focused on students or drivers in controlled and pre-prepared environments or within hospital settings, our research stands out by being conducted in an industrial setting, directly within the working environment of the participants. Furthermore, no prior study has specifically examined the impact of music on assembly workers, particularly female workers. In the assembly industry, given the monotonous and repetitive nature of the work, the significance of cognitive ergonomics alongside the repetitive physical tasks and worker fatigue has been underscored. Previous research has highlighted the importance of music in reducing stress and fatigue. Our study aims to investigate the effects of music as a favorable auditory stimulus on both the fatigue and mental workload of workers. Simultaneously, we explore its influence on sensory perception, concentration, and task performance. By implementing strategic management interventions, we aim to enhance cognitive performance while concurrently alleviating stress and fatigue among workers, particularly female workers. Since assembly work is an activity that, in addition to causing physical fatigue, also leads to mental conflict and fatigue, imposing a cognitive load on individuals, this study aims to prevent work-related pressure and mental damage through early intervention. By doing so, we seek to mitigate the cognitive strain and enhance awareness of these activities. This approach is intended to contribute to the enhancement of well-being and the improvement of overall health within the work environment. Through the management of cognitive function and the reduction of stress and fatigue, we hope to take a step towards fostering a healthier and more positive working environment for all, with a special focus on the well-being of female workers.

The paper is structured as follows: Section 1 introduces the iterative process of conducting a literature review, outlines the subject of the study, and presents the general framework of the article. Section 2 details the research implementation method, including the introduction of tests and devices used. Section 3 examines the study’s results and findings. Section 4 interprets the findings, compares them with previous research in the field, and discusses the limitations of the present study. This section also provides suggestions for future researchers to achieve more comprehensive and improved results. Section 5 discusses the overall conclusions of this research.

## 2. Materials and methods

### 2.1 Study design

The current study is a cross-sectional investigation conducted among female assembly workers in the medical equipment manufacturing industry (specifically, Infusion Set) in Isfahan city. In this industry, 92 female assembly workers engage in 8-hour shifts within the assembly work unit. For participant selection in this study, entry criteria were established, including abstaining from consuming any caffeine within 8 hours before the test, being right-handed to mitigate the impact of dominant hand effects, having sufficient rest and sleep the night before the test, maintaining general health, and, for female participants, not being in their menstrual period. Also, Exclusion criteria included consuming any caffeine within 8 hours before the test, being left-handed, not having sufficient rest and sleep the night before the test, poor general health, being in their menstrual period, and unwillingness to participate in the study. After applying these criteria, 11 individuals were excluded, and the study proceeded to the implementation stage with 81 participants.

**Fig 1:**
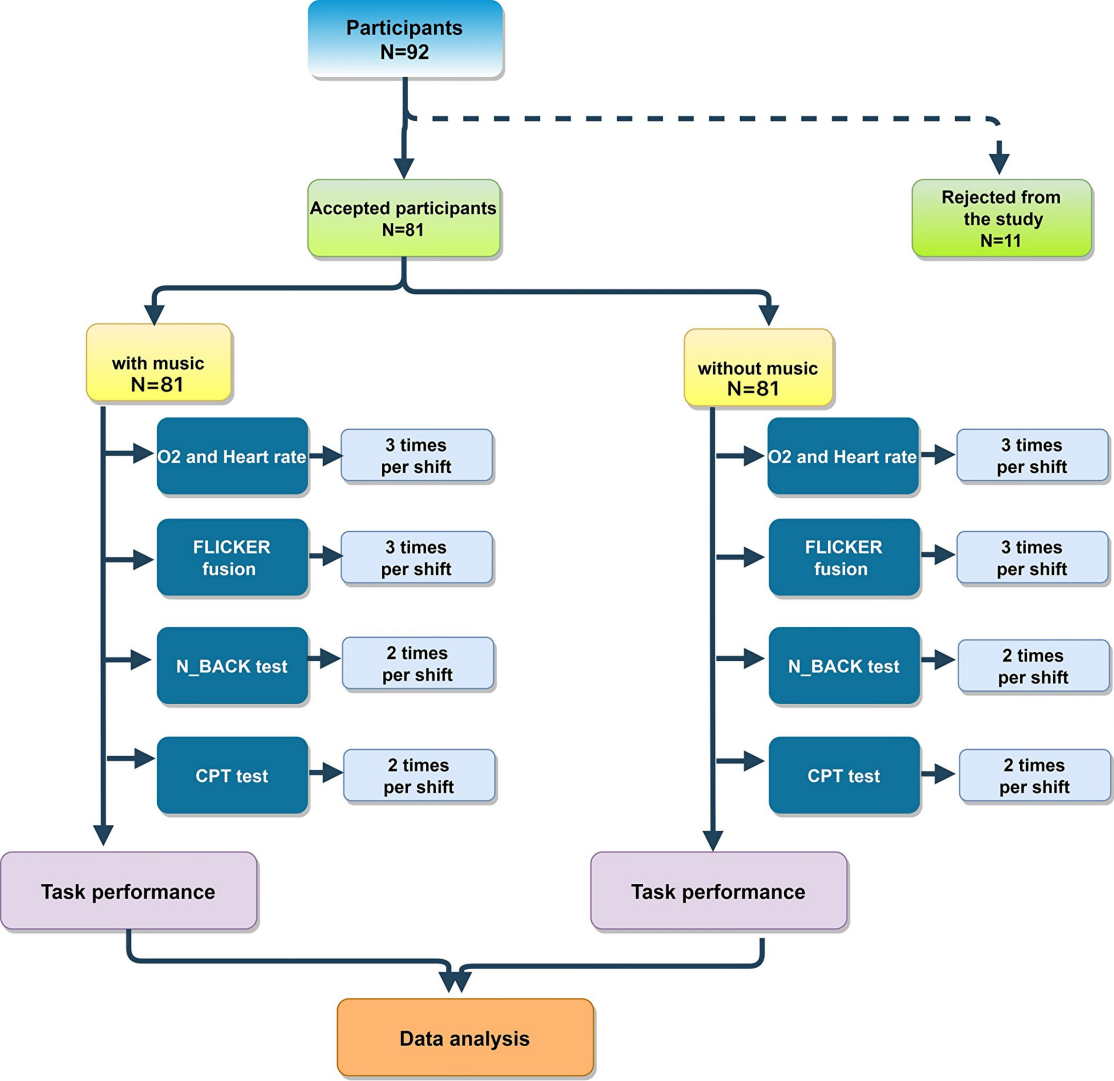
Study flow diagram.

Data were gathered through direct observation of the work process, interviews with both participants and the supervisor of the work unit, as well as the administration of questionnaires and cognitive tests (See S1 Data). The recruitment lasted from Thursday, 3^st^ August 2023 to Thursday, 14^st^ December 2023. The research proceeded through the steps illustrated in Fig 1.

Sampling was done by census. According to the study by Taheri et al. [13], which investigated the effect of music on cognitive performance, the mean and standard deviation of the hand skill score in the first group (without music) were 16.5±1.74, and in the second group (with music), the mean and standard deviation were 17.75±1.84. By calculating the effect size of 0.69 based on these parameters, the minimum sample size required for the present study, considering a type 1 error of 0.05 and a test power of 80%, was determined to be at least 20 participants per group using G*Power software and paired t-test analysis.

In the process of executing the research, instrumental classical music (specifically Mozart) was utilized via the speaker system within the production unit. In consideration of the potential hearing risks associated with sound levels exceeding 70 dB [34], as well as findings from a review study conducted by Dalton and Behm [11], which suggest that music within the 55–65 dB range may lead to optimal performance, the music in this study was played at the specified sound pressure level.

The general stages of research implementation are as follows:

- Gathering Participants’ Demographic Information: Collecting basic demographic details of the participants.
- Assessing Fatigue and Sleepiness: Evaluating fatigue and sleepiness using the Flickr Fusion device.
- Evaluating Cognitive Function: Measuring cognitive status with the CPT and N-back cognitive tests.
- Monitoring Physiological Indicators: Measuring blood oxygen levels and heart rate using a pulse oximeter.
- Assessing Task Performance: Measuring task performance by counting the number of quality products assembled and collecting quality control data from the industry.

The participants’ levels of mental fatigue and sleepiness were assessed using the Flickr Fusion device at the beginning, middle, and end of the work shift. Additionally, blood oxygen levels and heart rates were measured at the same intervals using a pulse oximeter. Cognitive tests, specifically the Continuous Performance Test (CPT) [13]. and active memory test (N-Back), were administered at the participants’ workstations at the beginning and end of the work shift. Lastly, task performance evaluation was conducted by tallying the number of assembled products meeting quality standards set by the industry’s quality control unit. It is noteworthy that all mentioned tests were conducted in two conditions: with music and without music.

### 2.2 Infusion set pats assembly industry

**Fig 2:**
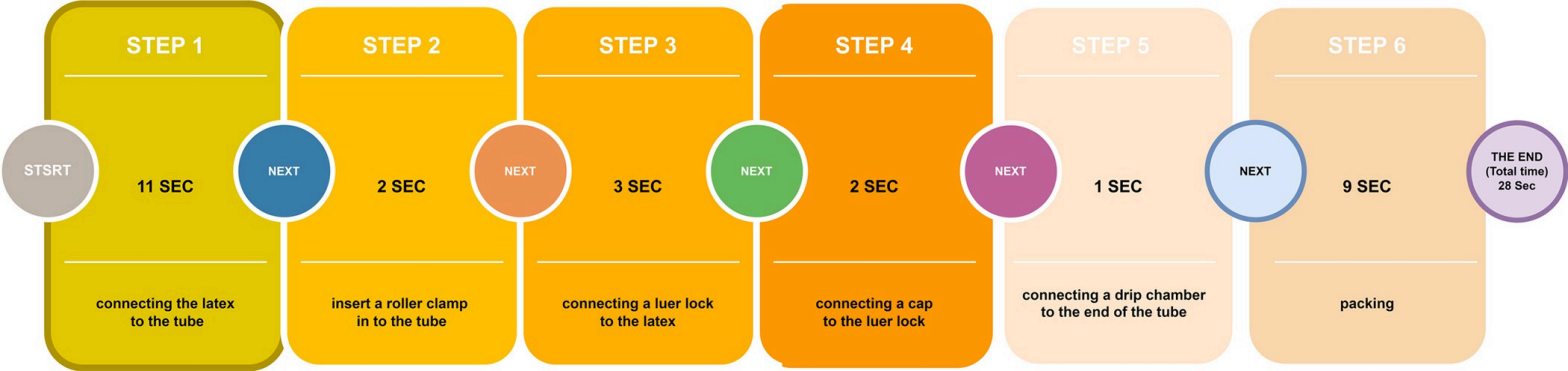
Production process.

This medical equipment manufacturing company was founded in 2009 and commenced operations in 2010. In recent years, it has endeavored to meet the demands of the health and treatment sector by expanding its cleanroom production unit. This expansion facilitated the production of high-quality infusion sets that adhere to international standards. Within this industry, individuals engage in the assembly and packaging of components for incoming infusion sets from the injection department during an 8-hour work shift. The average time for each step is depicted in Fig 2.

### 2.3 Evaluation and intervention tools

All tests and evaluations conducted in this research were performed in both conditions—with and without music intervention—on the same group of 81 assembly personnel from the specified industrial unit over the course of 2 consecutive working days.

#### 2.3.1 Music

A non-lyrical piece of classical music served as the intervention for this study and was played through the loudspeaker at a consistent volume for all participants during the intervention phase. In the music-playing condition, tests were conducted at the beginning and end of the work shift to explore the impact of music on the cognitive and task performance of individuals throughout the entire work shift. Considering the serene and quiet nature of the study environment within the target industry, a frequency range of 55–65 dB (including both the frequency of the music and background noise) was employed. The equivalent sound pressure level was measured using a calibrated sound level meter (CEL 110/2 calibrator, manufactured in the UK, model TES 1351B) in accordance with the ISO 9612–2009 standard [35].

#### 2.3.2 Assessment of level of sleepiness using the Flicker Fusion test

In this study, participants’ sleepiness was assessed using the Flicker Fusion device prior to engaging in the work tasks. Historically, this tool has been employed in various studies to evaluate individuals’ fatigue levels [36]. Over the years, measuring the eye’s ability to detect frequency, known as the Flicker Fusion Test, has been widely adopted as an indicator of fatigue severity. In this method, a flickering lamp with an adjustable flashing frequency is employed. The subject is positioned facing the lamp, and the flashing frequency is gradually increased, prompting the subject to indicate when they perceive a continuous light from the lamp [37]. In this method, the examiner observes the flashing light of the device at three stages—beginning, middle, and end of the work period—in standard conditions. Each time the light appears as a continuous beam, the examiner records the number displayed on the device. The recorded number indicates that a higher value corresponds to lower levels of fatigue and sleepiness, while a lower value indicates higher levels of tiredness and drowsiness.

#### 2.3.3 Evaluation of working memory using active memory test (N-Back)

The N-Back test is primarily utilized to assess working memory, and in this study, it will be employed for the evaluation of working memory. The N-Back test tasks individuals with measuring executive function, a commonly used metric in neuroimaging studies designed to stimulate brain function. Introduced by Kirchner in 1958 [38], this tool evaluates the ability to process, select, and store information within a very brief time frame. During a five-minute session, a total of 120 digits are displayed at intervals of 1500 milliseconds in the center of the screen. An N-Back test can involve 1 to N steps. For instance, in a two-step N-back test, during the first stage (1-back), participants are instructed to promptly compare two consecutive digits appearing on the screen. If the two numbers are the same, participants press the designated answer button on the specialized keyboard. In the second stage (2-back), participants are required to press the designated button if the current stimulus matches the one presented two stimuli before. Ultimately, the response time and the average number of correct answers are recorded as dependent variables in this test. The test output includes the completion response time and the count of correct responses [39].

#### 2.3.4 Evaluation of sustained attention using Continuous Performance Test (CPT)

The test, initially designed in 1956 by Rosvold et al., was originally employed to measure cerebral lesions. It found application as a diagnostic tool for attention deficit hyperactivity disorder (ADHD) in children during the 1990s and has since become widely acknowledged as the predominant laboratory instrument for evaluating sustained attention. Its primary objective is to assess sustained attention, while a secondary purpose is to gauge impulsivity or impulse control. During the test, participants are required to focus on a relatively straightforward series of visual or auditory stimuli for a specified duration. When the target stimulus appears, the participant must respond by pressing a designated key. In this study, a visualized and standardized version of the test was adapted for the Iranian population, encompassing three variables: response errors, deletion responses, and reaction time. Each stimulus is displayed for 200 milliseconds with a 1-second interval between stimuli. The entire test, including the training phase conducted to familiarize the volunteer with the expectations before the main stage, lasts for 200 seconds [13, 40]. The Continuous Performance Test (CPT) was utilized to measure attention errors and sustained attention. In the present study, a validated Persian version of this instrument was used. The test comprises 150 stimuli displayed on the screen, and participants are required to press the spacebar on their keyboards whenever the number "4" appears. Each stimulus is presented for 150 milliseconds (ms), with a 500 ms interval between consecutive stimuli. The dependent variables recorded include the number of correct responses, omission errors, commission errors, and response time (in milliseconds) [39].

#### 2.3.5 Measurement of blood oxygen level and heart rate

A pulse oximeter (ChoiceMMed pulse oximeter, CN356 model) was employed to assess heart rate and blood oxygen levels at the beginning, middle, and end of shiftwork in this study.

#### 2.3.6 Evaluating productivity through task performance measurement

At the end of the work shift, task performance was assessed in two test modes (with music and without music) by tallying the number of quality products assembled and gathering information from the quality control unit of the studied industry.

### 2.4 Data analysis

Descriptive statistical indices (frequency, percentage, mean, and standard deviation) were employed for data analysis. The normality of the data was assessed using the Kolmogorov-Smirnov (K-S) sample test. Based on this assessment, paired t-tests and the non-parametric Wilcoxon test were applied to each of the N-Back and CPT indices to compare data before and after playing music. The data were analyzed using IBM SPSS software, version 22, developed by SPSS Inc. in the USA. The significance level in this study was set at less than 0.05 (P-value ≤ 0.05). Additionally, all variables in the present study exhibited a normal distribution.

### 2.5 Ethics approval

This study was approved as a research project in the ethics committee of Isfahan University of Medical Sciences with code number IR.MUI.RESEARCH.REC.1402.107 and was performed in accordance with Declaration of Helsinki. In the present study all participants were above 18 years old and signed an informed consent form prior to taking part in the study. Before starting the measures, the necessary explanations about this study were given to the workers and their written consent was obtained to participate in this study. Workers were also assured that their personal information would be kept confidential. All methods in our study were performed in accordance with the guidelines and regulations approved by the Ethics Committee of Isfahan University or Medical sciences.

## 3. Results

### 3.1 Descriptive indices of study variables

The mean age of participants in this study was 33.09 ± 7. Additionally, the mean work history among participants was 1.3±0.2 years. Furthermore, 56.7% of the individuals were married. According to the findings presented in Table 1, the average cognitive performance, working memory, and sustained attention showed a decrease at the beginning of the working hour in the presence of music. However, these measures increased by the end of the working hour when music was played, and a simultaneous bell was heard throughout, resulting in an overall enhancement of cognitive performance.

**Table 1:** Descriptive indices of cognitive performance component scores in conditions of both playing and not playing music.

Variable	Working condition	Beginning of working hours		End of working hours		Total	
		mean	SD	mean	SD	mean	SD
Working memory	With music	926.53	137.51	897.73	110.34	912.13	114.09
	Without music	902.63	114.31	977.81	113.85	940.72	95.00
Sustained attention	With music	1.59	1.43	1.07	0.97	1.33	1.09
	Without music	1.46	1.26	1.44	1.16	1.44	1.07
Alertness	With music	1.26	1.19	0.92	0.91	1.09	0.96
	Without music	1.31	1.13	1.64	0.83	1.47	0.88
Cognitive function	With music	929.38	137.57	899.72	110.64	304.85	38.07
	Without music	906.29	114.69	980.90	113.69	314.55	31.57

Furthermore, the results presented in Table 2 indicate a slight decrease in the average of all components at all working times in the condition without music.

**Table 2:** Descriptive indices of job fatigue component scores in conditions of playing and not playing music.

Variable	Working condition	Beginning of working hours		Middle of working hours		End of working hours		Total	
		mean	SD	mean	SD	mean	SD	mean	SD
Sleepness	With music	37.92	3.00	40.11	2.73	39.02	2.72	39.02	2.59
	Without music	37.73	2.27	38.60	2.23	38.38	2.33	37.90	2.00
Heart rate	With music	80.42	7.44	79.52	6.94	79.57	7.19	79.83	7.03
	Without music	78.62	5.81	78.73	5.69	79.25	6.12	78.86	5.50
Blood oxygen	With music	95.76	1.26	95.75	1.08	94.95	9.75	95.48	3.39
	Without music	95.77	1.39	95.43	1.25	95.04	1.18	95.41	1.06
job fatigue	With music	214.11	7.67	215.38	7.71	213.54	12.12	213.34	8.11
	Without music	212.12	6.56	212.76	6.29	211.67	6.81	212.18	6.15

### 3.2 Investigating the relationship between playing music and working memory

The findings presented in Table 3 highlight variations between the conditions of playing and not playing music at the beginning and end of working hours. Additionally, notable differences in working memory and response time are observed at the end of the work shift, with significant improvements when music is played. The results at the end of the shift demonstrate a significant disparity between the music and no-music conditions, showcasing improvements attributed to the presence of music. Furthermore, the time required at the end of the work shift significantly reduces in the music mode. Interestingly, in the music mode, there is a significant difference in results between the beginning and end of the shift, indicating overall improvement. However, the time factor does not exhibit a significant difference. In contrast, in the condition without playing music, there is a significant distinction in results between the beginning and end of the shift, with a decrease in correctness rate. Moreover, there is a significant difference in response time of the test, indicating an increase in time for responding and concluding the test.

**Table 3:** The results of the relationship between playing music and working memory (N-BACK test).

Variable	Variable	Time of work shift	Working condition		p-value
			Without music (Mean±SD)	With music (Mean±SD)	
N-BACK	Working memory	Beginning of time	56.51±2.47	56.74±2.48	0.446
		End of time	55.25±2.09	58.29±2.09	0.001^*^
		*p-value (time of work shift)*	0.004^*^	0.001^*^	
	Response time	Beginning of time	847.12±113.67	869.79±138.03	0.325
		End of time	922.57±113.17	839.43±110.29	0.001^*^
		*p-value (time of work shift)*	0.001^*^	0.100	

^*^. Significance level was considered as p-value < 0.05.

### 3.3 Examining the relationship between playing music and sustained attention

The findings in Table 4 indicate a significant difference in response time when playing music versus not playing music at the beginning of working hours. Moreover, playing music at the end of working hours influences omission error, commission error, and the number of correct answers. Additionally, in the absence of music, commission error and the number of correct answers differ between the beginning and end of the working hours.

**Table 4:** The results of the relationship between playing music and sustained attention (CPT test).

Variable	Variable	Time of work shift	Working condition			p-value
			Without music (Mean±SD)		With music (Mean±SD)	
CPT	Response time	Beginning of time	624.66±95.12		572.37±113.65	0.001^*^
		End of time	651.19±86.39		648.13±95.76	0.808
		*p-value (time of work shift)*	0.128		0.000**	
	Omission error	Beginning of time	1.46±1.25		1.59±1.43	0.655
		End of time	1.44±1.16		1.07±0.97	0.009^*^
		*p-value (time of work shift)*	0.893	0.024^*^		
	Comission error	Beginning of time	1.31±1.14		1.26±1.19	0.633
		End of time	1.64±0.83		0.92±0.91	0.001^*^
		*p-value (time of work shift)*	0.012^*^		0.070	
	Right answer	Beginning of time	147.38±1.54		147.13±2.02	0.288
		End of time	146.931.51±		147.99±1.45	0.001^*^
		*p-value (time of work shift)*	0.049^*^		0.001^*^	

^*^. Significance level was considered as p-value < 0.05.

In cases where music is played, response time, omission error, and the number of correct answers also exhibit differences between the beginning and end of the working hour. The duration of the CPT test significantly decreases in the music mode at the beginning of the work shift. In both the music and no-music modes, the duration of the test is longer at the end of the shift compared to the beginning. There is a significant difference in responses deleted at the end of the shift between the music and non-music modes, with a decrease in the music mode. CPT test errors at the end of the work shift significantly decrease in the music-playing mode, and correct answers show improvement in the end-of-shift music mode.

### 3.4 Investigating the relationship between playing music and the variables of sleepiness and mental fatigue, heart rate and blood oxygen

In the results presented in Table 5, it is evident that, when music is not playing, Flicker fusion test results and blood oxygen levels vary at different working times. Conversely, when music is playing, Flicker test and heart rate show differences at different working times. Specifically, at the beginning of the working hour, playing music has an effect on heart rate. In the middle of the working hour, playing music affects Flicker test and oxygen levels, and at the end of the working hour, playing music affects Flicker fusion Test results.

**Table 5:** Correlation between playing music with the variables of sleepiness and mental fatigue, heart rate and blood oxygen.

Variable	Time of work shift	Working condition			p-value
		Without music (Mean±SD)		With music (Mean±SD)	
Flicker fusion test	Beginning of time	37.73±2.26		37.93±3.00	0.645
	Middle of time	38.59±2.23		40.11±2.73	0.001^*^
	End of time	37.38±2.33		39.02±2.72	0.001^*^
	*p-value(time work)*	0.001^*^		0.001^*^	
Heart rate	Beginning of time	78.62±5.81		80.42±7.44	0.001^*^
	Middle of time	78.73±5.69		79.52±6.94	0.122
	End time	79.25±6.12		79.57±7.19	0.612
	*p-value(time work)*	0.240	0.005		
Blood oxygen	Beginning of time	95.77±1.39		95.76±1.26	0.930
	Middle of time	95.43±1.25		95.75±1.08	0.016^*^
	End of time	95.04±1.19		94.95±9.75	0.937
	*p-value(time work)*	0.001^*^		0.460	

^*^. Significance level was considered as p-value < 0.05.

The findings indicate that the degree of fatigue and sleepiness, as measured by the flicker fusion tool, significantly differs in the middle and end of the work shift. It is higher in the music-playing mode than in the non-music playing mode, indicating a decrease in sleepiness and fatigue. In the music playback mode, the degree of fatigue and sleepiness exhibits a significant difference, being higher in the middle of the shift and decreasing by the end of the shift compared to the middle. However, its value remains higher than at the beginning of the shift. In the case of not playing music, there is a significant difference in the degree of fatigue and sleepiness during the measured hours, with higher levels in the middle of the work shift and a decrease at the end, indicating an increase in fatigue and sleepiness towards the end of the shift.

A significant difference in heart rate exists between the two modes of playing music and not playing music at the beginning of the work shift, with a higher rate in the music playing mode. In the music playing mode, there is a significant difference in heart rate at the beginning, middle, and end of the work shift. In the middle of the shift, it is lower than at the beginning, and at the end of the shift, it slightly increases compared to the middle, but it remains lower than at the beginning.

There is a significant difference in the blood oxygen level of the participants between the two modes of playing music and not playing music in the middle of the work shift, with higher levels in the mode of playing music. In the state of not playing music, there is a significant difference in the blood oxygen level between the beginning, middle, and end of the work shift. As the shift progresses towards the end, the blood oxygen level decreases.

### 3.5 Investigating the relationship between music and productivity

**Fig 3:**
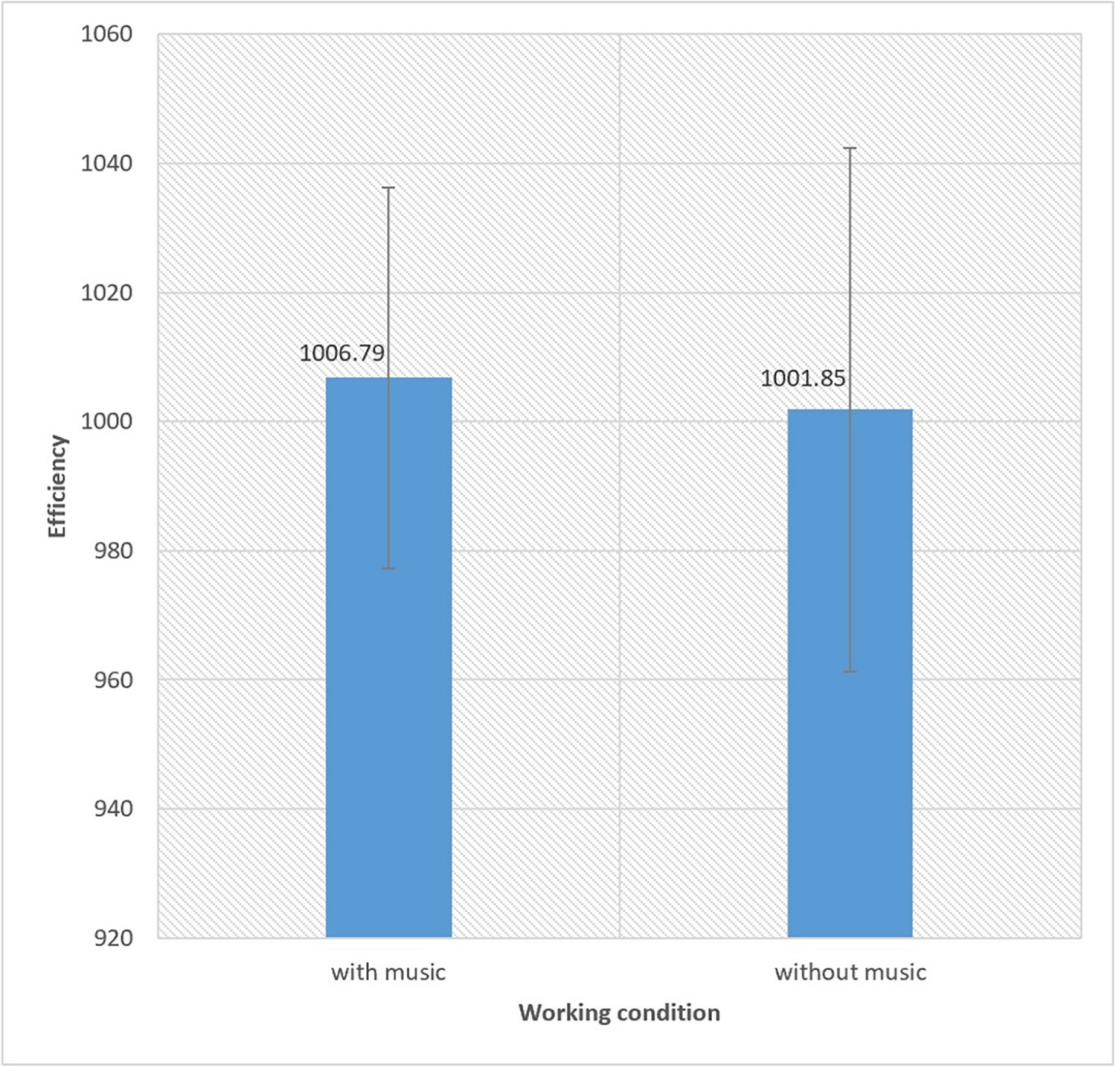
The results of the relationship between productivity and playing music.

Fig 3 indicates a significant difference in productivity between the conditions of playing music and not playing music, with the level of productivity being higher in the music-playing mode compared to the non-music playing mode at a significance level of 0.005 (P-value = 0.005).

## 4. Discussion

The present study aimed to assess the impact of music on the cognitive performance of female assembly operation operators in a medical equipment manufacturing company. With an increasing presence of women in industrial settings, there is a growing need for studies to enhance the working conditions of female workers. The assembly operation involves repetitive tasks that lead to physical and mental fatigue. This research sought to examine the effects of music intervention on cognitive performance, sleepiness, blood oxygen levels, and heart rate among the participants. The goal was to contribute to the improvement of working conditions for women in this field without compromising the quality of their work. The study included 92 female workers in the assembly unit for infusion set parts at a medical equipment production facility. After applying entry and exit criteria, 81 individuals proceeded to the testing phase. Participants had the option to withdraw from the study at any stage if they chose not to continue their cooperation.

The N-Back test results clearly indicate that music had a positive impact on performance. In the absence of music, the test duration increased at the end of the work shift. These findings support the hypothesis that music enhances the working memory of participants in a workplace setting. This aligns with the outcomes of a study conducted by Taheri et al., where they investigated the impact of music on working memory in a gender and personality type (introvert/extrovert) context. Their results revealed a significant improvement in working memory with an increase in correct answers and a decrease in response time when music was played [13]. Another study by Chew et al. involved 165 undergraduate students performing math tasks, reading comprehension, and word memory tasks while exposed to familiar or unfamiliar music, a foreign or first language, and no music. The results demonstrated that word memory tasks performed under the influence of music, especially familiar music, showed significant improvement in scores [41]; which are in line with the results of the present study. However, the study conducted by Christopher and Shelton aimed to investigate the effect of music on the performance of a group of students at the University of Tennessee at Chattanooga and its correlation with working memory capacity. The results of this research, which explored the impact of music on memory while solving math problems, revealed no significant relationship between music playback and student performance or its association with working memory capacity [42]. This lack of correlation may arise from the fact that the study did not directly measure the effect of music on working memory. Instead, it focused on reading comprehension and math calculations performance with music intervention, examining the relationship between overall performance and working memory based on the results of music intervention. In another study using a crowdsourcing approach to evaluate participants’ stress and cognition, as well as investigating the impact of 30 days of mental focus and music intervention, the results did not demonstrate a significant effect of music on working memory in the n-back test. Consequently, it was concluded that music does not appear to influence working memory [43]. The discrepancy between the findings of this study and the present study could be attributed to the short duration of music playback during the test.

The results of the CPT test, comparing the two conditions of playing music and not playing music, demonstrated a clear improvement in results with music, leading to a reduction in test errors. However, no significant difference was observed in the duration of the test. This portion of the present study supports the hypothesis that music enhances participants’ sustained attention in the workplace. Contrary to these findings, Taheri et al.’s research reported that music had no significant effect on sustained attention. Their study indicated that although music decreased the number of incorrect answers, it increased the number of unanswered trials and reaction time. However, none of these differences reached statistical significance [13]. This discrepancy can be due to a change in the environment of the test, the gender difference in the mentioned article, or the lack of attention of the participants to the test.

In another study, participants performed cognitive tasks either in silence or with music of varying complexity and volume. The study revealed that music might induce attentional conflicts, especially in tasks requiring multiple sources of attention, leading to divided attention and increased distraction, making the work more challenging. Therefore, the impact of music on performance is contingent on factors such as the type of music, the nature of the task, and the individual performer [44]. This discrepancy may be attributed to differences in task complexity or laboratory conditions. However, the study by Herleka and colleagues yielded results consistent with the present study. In their research, post-test scores of Indian and Malaysian participants in the Symbol Digit Modalities Test (SDMT), a measure of attention performance, were higher in the music group compared to the control group (no music). Specifically, Malaysian participants in the music group outperformed other groups in the attention test [45].

This study continued its examination by assessing the level of fatigue and sleepiness, employing the flicker fusion tool. In the music-playing mode, there was an increase in the degree of fatigue and sleepiness compared to the non-music-playing mode, signifying a decrease in sleepiness and fatigue. Within the music-playing mode, the degree of fatigue and sleepiness exhibited a significant difference throughout the measured hours. Specifically, it peaked in the middle of the shift and decreased by the end of the shift compared to the midpoint, yet it remained higher than at the beginning of the shift. Conversely, participants in the non-music-playing mode showed an escalating level of fatigue and sleepiness towards the end of the shift. A study investigating the impact of music tempo on driver fatigue and attention quality in a controlled environment yielded results in line with this aspect of the present study. The study demonstrated that music has the potential to mitigate driver fatigue, and the tempo of the music plays a role in its effectiveness [14]. Additionally, the outcomes of this section align with the research conducted by Shibani et al. Their study explored the effects of high-speed pop music and Iranian classical music on psychological and physiological drowsiness in drivers. The findings revealed that listening to both types of music enhanced driver performance and reduced drowsiness, suggesting that higher-speed music during driving can alleviate fatigue [34]. Wang and Zhendong conducted a study on drivers using EEG signals to analyze driving fatigue and prevent associated dangers and injuries. The results of their study supported the notion that music contributes to reducing driver fatigue [46]. As a result, in the mentioned studies like the present study, it is evident that the positive effect of music on the reduction of fatigue and sleepiness of the participants, it is possible to infer the effectiveness of playing music in the context of reducing fatigue.

The comparison of heart rate measurements obtained with the pulse oximeter device between the modes of playing music and not playing music revealed a significant difference in the music-playing mode. Specifically, the heart rate exhibited variance at the beginning, middle, and end of the work shift. In the middle of the shift, it was lower than at the beginning, and at the end of the shift, it slightly increased compared to the middle, yet remained lower than at the beginning. Conversely, in the no-music-playing mode, there was no significant difference in heart rate across the beginning, middle, and end of the work shift. These results align with findings from a study conducted on dialysis patients, indicating that the average diastolic blood pressure and heart rate in the music intervention group were lower than the control group. This suggests that implementing music therapy during dialysis sessions can enhance patients’ comfort [47]; Therefore, in terms of the positive effect of music on heart rate, it is the same with the present study. Changes in heart rate have been explored and reported in various studies; for instance, Knight’s study demonstrated a decrease in heart rate during exposure to relaxing music [30]; which in this case supports the results of the present study. But another study involving Iranian drivers found that the average heart rate increased when listening to music while driving compared to driving without music [34]. The results of this study diverge from the current study, potentially due to variations in participants’ working environments or the specific type of music played. It’s worth noting that Blood & Zatorre found that stimulating music increases heart rate [32]. The discordance in findings between this research and the present study may stem from differences in the genre and style of the music utilized. Blood oxygen levels of participants were measured using a pulse oximeter in both the music and non-music conditions. A significant difference in blood oxygen levels emerged between the two modes during the middle of the work shift, with an increase observed in the music-playing mode. No significant difference was noted at the end of the work shift. Furthermore, in the absence of music, a notable difference in blood oxygen levels surfaced across the beginning, middle, and end of the work shift. Oxygen saturation tends to induce a relaxed state [48], and the decline in blood oxygen levels at the end of the shift without music may be indicative of heightened work-related stress. A study exploring the impact of background music on relaxation and work efficiency, based on survey data from university students, found that instrumental music without lyrics had the most significant effect on relaxation and increased oxygen saturation [48], aligning with the findings of the present study. In another investigation, aimed at evaluating the effects of music therapy during or after surgery on stress and immune responses during and after general anesthesia, the researchers did not observe a significant difference in oxygen saturation with music therapy [49]. Additionally, a randomized controlled trial involving 40 patients explored the impact of single-session music therapy on anxiety and vital parameters in hospitalized COVID-19 patients. The results indicated that one session of music therapy improved oxygen saturation and significantly reduced anxiety [50].

In this study, the task performance of participants, involving the assembly of infusion set parts and subsequent packaging, was assessed in two conditions: playing music and not playing music. The evaluation was based on the count of assembled and packaged high-quality products, revealing an enhanced work productivity in the music-playing mode compared to the non-music-playing mode. Taheri et al. also conducted research on students’ manual skills using the Two-arm coordination test, showing that music improves skill performance, particularly in work environments where manual skills are crucial [13]. These findings align with the results of the present study. In a 2011 study conducted in an Indian hospital with 100 participants, including nurses, surgeons, and anesthesiologists working in the operating room, the aim was to investigate whether music could enhance the efficiency of medical personnel in the operating room. The results indicated that music indeed contributed to improving the efficiency of medical personnel in the operating room [25]. Similarly, a 2022 study in Taiwan focused on nurses and explored the impact of various types of noise and music on anxiety, mental workload, and awareness during surgery. The study concluded that music played at 60 dB during surgery could be a practical solution to mitigate the negative effects of additional noise, thereby improving nurses’ performance [24]. However, it’s important to note that conflicting results exist. One study found that the performance of surgeons was actually worse in a musical environment, particularly during simulated endoscopic surgery and a modified speech-in-noise test [51]. On the other hand, research by Jamshidzad et al. aimed to investigate the effect of music type on movement coordination performance in introverted and extroverted students, demonstrating that listening to music improved and increased performance speed [52].

Other studies have been conducted in recent years on the effect of music on cognitive performance, sustained attention, and work-related stress, which have similar results to the present study [43, 53, 54]. For example, we can refer to research conducted in 2020 with the aim of investigating whether music intervention and auditory stimulation can reduce the negative effects of mental fatigue on subsequent cognitive processing. The participants were tested in 5 stages, and the results showed that the music group had the least effect on mental fatigue and its effect on the success rate, while the performance of the control group and the novice mindfulness group decreased with mental fatigue [53]. Also in 2019, researchers conducted a study with a university student population to investigate whether laboratory evidence of mind wandering could be reduced through in situ auditory stimulation intervention. The results showed that 15 minutes of binaural hearing led to a significant reduction in mind wandering, while the control group did not show any differences [54].

To further confirm the positive effect of music in reducing errors while doing work, we can look at the study of Eva Nadon et al. in 2021. The purpose of this research was to investigate the relaxing and stimulating effect of background music on selective attention. For this purpose, 46 healthy adults performed a predetermined task in five different sound environments: soothing music, stimulating music, noise-matched soothing music, noise-matched music, and silence. Interestingly, the results showed that task errors were reduced by playing soothing music [55].

Research was also conducted in recent years with the aim of investigating the effect of music therapy on stress levels and the risk of burnout among operating room staff. This was a pre-experimental study involving the staff of the urology and maxillofacial surgery operating room at Sahl Sous University Hospital (Tunisia) over a period of six weeks. The study consisted of three stages. The first session was the initial assessment of the stress level with a pre-defined survey; the second session consisted of three music therapy sessions per day during one month; and the third was the re-evaluation of the stress level immediately after the intervention. Finally, the findings led to the conclusion that music therapy is an innovative approach that seems to reduce the stress of operating theater staff [56].

In 2023, researchers investigated the effects of music-induced positive and negative emotions on spatial working memory performance using a within-subjects design. In addition, they simultaneously recorded the physiological responses of 78 participants while listening to musical stimuli. Overall, these findings indicated that positive emotion-inducing music can enhance visuospatial working memory performance [57]. Another study in 2023 with 40 third-year dental students aimed to investigate whether listening to slow background music can reduce students’ stress when learning how to perform cavity preparation and restoration in a simulation laboratory, and the effect of slow background music on the quality and time spent in It was done during cavity preparation. Finally, music reduced stress and increased motivation to learn and practice. Communication in class went well despite the music. The use of time and the quality of cavity preparation increased. The results showed that music has beneficial effects on teaching and practicing dental skills [58].

The current study focused on female employees, allowing for potential future comparisons with male employees. In addition to examining the impact of music on cognitive performance, memory, and attention, we explored its intervention effects on sleepiness and physiological parameters such as heart rate and blood oxygen levels. In past studies, the effect of all these things on a group of participants has not been investigated at the same time, which can be a good value for future studies. Also, in most of the studies, the location of the test was predetermined and it was done under controlled conditions. In this study, we were able to achieve results in real work conditions by conducting research in the work environment and under normal conditions. These additional investigations contribute valuable insights to the existing research in this field. However, certain limitations should be acknowledged, including the omission of participants’ music preferences and the exclusion of various music genres. Specifically, classical music was the sole intervention used. Additionally, the study did not delve into the influence of participants’ personality types. It’s noteworthy that the research exclusively concentrated on employees engaged in assembly activities, and the diversity of job roles was not taken into account and it was not possible to determine a specific age range. Another limitation of this study was its small sample size, as it was conducted in a single industrial unit. Conversely, since this study was conducted exclusively in Isfahan, Iran, it did not account for the participants’ cultural characteristics.

It is recommended that future research on this topic include male participants to allow for gender-based comparisons of the results. Additionally, researchers should consider exploring multiple, diverse cognitive activity models concurrently. Investigating various genres of music in the work environment, as well as incorporating participants’ musical preferences, could provide valuable insights and contribute to more comprehensive findings in future studies. Also, Future research could improve sample size by including multiple industrial units simultaneously. Furthermore, Future research could address cultural characteristics by including participants from diverse cities with varying cultural backgrounds to assess how these differences might impact the study’s results.

## 5. Conclusions

The present study showed that music can have a positive effect on the working memory of female workers. In general, it can be said that programs that require memory or jobs that require active memory should be run at the same time as playing music to improve their cognitive performance. Also, the positive effect of music on manual skills, reduction of mental fatigue and sleepiness was determined in this study. Conclusion about the effect of background music on attention as well as parameters of heart rate and blood oxygen level need more research. In this research, music improves skill performance and playing music in work environments where a person’s task is related to manual skills seems desirable. However, the effect of music on the ergonomic abilities of workers with male gender and the role of personality type on the effect of music has not been investigated and needs more research. Based on the study’s findings, the following practical recommendations are proposed:

- Integrate music into work environments where active memory is needed to enhance cognitive performance.
- Implement background music in workplaces where manual skills are required, as it may enhance skill performance, efficiency, and productivity.
- Employ music to help alleviate mental fatigue and sleepiness, which can maintain alertness and reduce errors.
- Explore how background music affects attention, heart rate, and blood oxygen levels in more depth.

By adopting these recommendations, organizations can potentially boost worker performance, well-being, and productivity through the strategic use of music in the workplace.

## Supporting information

S1 Data Final data used in the study. (XLSX)
